# Effect of low-dose esketamine combined with labor analgesia on postpartum depressive symptoms following spontaneous labor: a randomized controlled trial

**DOI:** 10.3389/fmed.2026.1722131

**Published:** 2026-02-19

**Authors:** Jianxin Gao, Lan Dai, Lulu Qin, Baiqing Duan, Dan Peng, Xi Huang, Liyunjian He, Jingni Zou, Qin Zhou, Dai Zhou

**Affiliations:** 1 Hunan Provincial Key Laboratory of Regional Hereditary Birth Defect Prevention and Control, Changsha Hospital for Maternal & Child Health Care Affiliated to Hunan Normal University, Changsha, Hunan, China; 2 School of Public Health, Hunan Normal University Health Science Center, Changsha, China

**Keywords:** epidural analgesia, esketamine, labor analgesia, postpartum depression, spontaneous labor

## Abstract

**Background:**

Postpartum depression (PPD) poses significant risks to maternal and infant well-being. This study aimed to assess the effect of low-dose epidural esketamine combined with labor analgesia on postpartum depressive symptoms and pain scores in women undergoing spontaneous labor.

**Methods:**

A randomized controlled trial was conducted with 299 participants assigned to three groups: Group A (esketamine 0.5 mg/kg + labor analgesia), Group B (labor analgesia alone), and Group C (no analgesia). The primary outcome was the Edinburgh Postnatal Depression Scale (EPDS) score at 42 days postpartum. Secondary outcomes included Visual Analogue Scale (VAS) pain scores and serum hormone levels.

**Results:**

Overall, EPDS scores were low across the cohort. At 42 days postpartum, no statistically significant difference in EPDS scores was observed between the esketamine group and the analgesia-only group (Group A: 1.97 ± 1.74 vs. Group B: 2.01 ± 1.68). The mean difference was −0.03 (95% CI: −0.46 to 0.39, *p* = 0.88). The incidence of screen-positive depression (EPDS ≥10) was also comparable among groups (4% vs. 2% vs. 6%). Longitudinally, all groups showed temporal improvements in depressive symptoms and pain scores, which coincided with the physiological decline in estrogen and rise in cortisol levels post-delivery.

**Conclusion:**

In this low-risk cohort with low baseline depressive symptoms, the addition of low-dose esketamine to labor analgesia did not result in a significant reduction in EPDS scores at 42 days compared to labor analgesia alone. These findings suggest that the potential benefits of esketamine might be limited in populations with a low risk of PPD, and future studies focusing on high-risk groups are warranted.

**Clinical trial registration:**

https://www.chictr.org.cn/showproj.html?proj=247161, identifier ChiCTR2500104037.

## Introduction

Postpartum depression (PPD) is a prevalent perinatal psychological disorder, with the incidence varying between 11 and 27% across different countries. Its symptoms include depression, sadness, and anhedonia (loss of pleasure) (1, 2). Given the significant risks associated with PPD, including harmful behaviors toward infants and oneself, as well as detrimental effects on family dynamics (3), researching early intervention measures is of paramount importance.

The etiology of postpartum depression is multifactorial, involving various psychosocial risk factors. These include pre-existing major depressive disorder, anxiety, stressful life events during pregnancy or the early puerperium, lack of social and financial support, and young maternal age—each of which has been associated with an increased risk of postpartum depression (4).

Recent studies have shown a close relationship between hormonal changes in pregnant women and the onset of postpartum depression (5). After the delivery of the placenta, there is a sharp decline in hormones such as estrogen and progesterone, which may contribute to the suppression of monoamine neurotransmitter synthesis, release, and metabolism. This results in a significant reduction in serotonin (5-HT) levels, which impairs synaptic transmission and contributes to depressive symptoms (6). A substantial body of clinical research has also confirmed the association between changes in cortisol levels and the development of postpartum depression. While the body typically self-regulates during the postpartum period, excessive adjustments can lead to hypocortisolism, triggering depressive symptoms (7). Contrary to the negative feedback mechanism of the hypothalamic–pituitary–adrenal axis, the release of cortisol promotes the secretion of placental corticotropin-releasing hormone (pCRH) (8). Therefore, the significant decrease in pCRH levels after the delivery of the placenta may also contribute to the onset of postpartum depression.

Labor pain has been identified as a significant risk factor for the development of postpartum depression, though this risk can be mitigated by the use of analgesic medications. In non-obstetric populations, the relationship between chronic pain and depression is well-documented, with evidence suggesting that pain can exacerbate depression, and vice versa, creating a cycle of worsening symptoms (9). Maternal labor pain is considered a stressor that can disrupt the endocrine system, potentially contributing to the onset of postnatal depression. Recent literature emphasizes that poor management of perinatal pain is associated with decreased maternal care and a higher risk of developing PPD (10). Therefore, effective analgesic interventions are crucial not only for physical comfort but also for protecting maternal mental health. Studies conducted both nationally and internationally have shown that effective management of labor pain with analgesics can lead to a reduction in postpartum depression scores, as measured by the Edinburgh Postnatal Depression Scale (EPDS) at 6 weeks postpartum (11–13).

Ketamine, an N-methyl-D-aspartate (NMDA) receptor antagonist, is commonly used as an intraoperative sedative and analgesic due to its low respiratory depressant effects, making it suitable for obstetric anesthesia. Beyond its anesthetic properties, ketamine also demonstrates anti-inflammatory and antidepressant effects. Ketamine and its enantiomer esketamine are increasingly recognized as “psychoceuticals”—agents that target specific neural circuits to treat psychiatric disorders (14). By modulating NMDA receptors, these agents promote neuroplasticity and offer rapid-acting therapeutic benefits for conditions like depression and anxiety (14). The antidepressant efficacy of ketamine was first identified in a randomized controlled clinical trial (15), with subsequent studies confirming that a single intravenous dose of 0.5 mg/kg ketamine can prevent and treat postpartum depression (16, 17). In an animal model of postpartum depression induced by chronic stress, ketamine was found to reverse behavioral deficits and improve abnormal Akt–mTOR signaling in rats exhibiting depression-like symptoms (18). Clinical research has also indicated that the use of ketamine during cesarean delivery can reduce the incidence of postpartum depression, as assessed by EPDS score (19).

Esketamine, the dextrorotatory isomer of ketamine, has a more potent antidepressant effect than racemic ketamine and can be used at lower doses with fewer side effects and a higher safety profile (20). Animal studies have shown that esketamine has a higher affinity for NMDA, opioid, and muscarinic acetylcholine receptors compared to ketamine, while its inhibition of serotonin receptors is only half that of ketamine (21). Esketamine’s clearance rate is significantly higher than that of racemic ketamine. Ketamine has been safely used in spinal anesthesia, and esketamine, with its higher receptor affinity, has been employed in intra-sacral anesthesia since 2004 (22).

In recent years, there has been growing interest in the potential role of labor analgesics and other anesthetics in the prevention and treatment of postpartum depression. Research suggests that epidural analgesia (EDA), a widely used method for pain relief during labor, may have a protective effect against the development of postpartum depression. A study found that the incidence of postpartum depression was lower among women who received EDA compared to those who did not (23). EDA has also been shown to reduce stress hormone levels, which could help mitigate symptoms of postpartum depression (24). General anesthetics such as propofol and ketamine have also been the focus of research in this area. Propofol has been found to possess antidepressant properties and is associated with a decrease in the incidence of postpartum depression (25). Ketamine and its enantiomer esketamine have shown promising results in the treatment of postpartum depression (26). However, the role of labor analgesics and other anesthetics in the treatment of postpartum depression is still a subject of debate. Some studies suggest that these medications may affect the endocrine and nervous systems during labor, potentially influencing the onset and progression of postpartum depression (27). Therefore, labor analgesics and other anesthetics appear to play a role in the prevention and treatment of postpartum depression, but further research is needed to fully understand their mechanisms of action and clinical efficacy.

Ketamine and its enantiomer esketamine are increasingly recognized for their rapid-acting antidepressant properties. However, their use requires careful consideration of safety and efficacy profiles. A recent review by Di Vincenzo et al. highlighted the importance of distinguishing clinical facts from myths regarding esketamine use in treatment-resistant depression, emphasizing the need for rigorous evidence in specific populations (28). Furthermore, emerging evidence suggests potential gender differences in response to esketamine, with studies indicating variations in suicidal and self-harming ideation reduction between males and females (29). Given these complexities, investigating esketamine’s role in the specific context of postpartum depression prevention is warranted.

In this study, we explore the efficacy of combining labor analgesia with epidural esketamine in the prevention and treatment of postpartum depression. Additionally, we investigate the molecular mechanisms underlying postpartum depression by analyzing the levels of estrogen, progesterone, serum 5-hydroxytryptamine, and serum cortisol, thereby providing new insights for the clinical management of postpartum depression.

## Methods

### Ethical approval

This study was approved by the Medical Research Ethics Committee of Changsha Hospital for Maternal and Child Health Care (No. 2020012-01). All participants provided written informed consent. The study was conducted in accordance with the guidelines set by the International Council for Harmonization of Technical Requirements for Pharmaceuticals for Human Use (ICH), the Declaration of Helsinki, and relevant national regulations. The study adheres to CONSORT guidelines for randomized controlled trials.

The trial was registered retrospectively at the Chinese Clinical Trial Registry (ChiCTR2500104037) due to an administrative oversight. However, the study protocol, including the primary outcome (EPDS) and analysis plan, was finalized and approved by the ethics committee prior to participant enrollment and data analysis to ensure the integrity of the study design.

### Research design

This was a randomized, controlled, three-arm clinical trial. The study enrolled women who delivered at Changsha Maternal and Child Health Hospital, affiliated with Hunan Normal University, between February 2020 and January 2022. Due to the distinct nature of the intervention (intravenous medication vs. no medication in the control group) and clinical logistics, this study employed an open-label design. Full blinding of participants and clinicians was not feasible. To minimize potential detection bias, outcome assessors responsible for the postnatal follow-up (EPDS and VAS collection) were not involved in the intrapartum clinical care. The estimated incidence of postpartum depression in Chinese women with a normal vaginal birth is 25% based on the EPDS score cutoff at 9/10, and 14% for women with an uncomplicated delivery. Due to the limited data available at the inception of this study regarding the specific effect size of esketamine on PPD, we powered the study to detect a clinically meaningful difference. Based on our preliminary clinical observations, we hypothesized a 50% reduction in the incidence of postpartum depression in the group receiving epidural ketamine analgesia, resulting in an incidence of 7%. With a statistical power of 80%, a significance level of *α* = 0.05, and an estimated 10% dropout rate, we calculated a sample size of approximately 110 participants per group.

### Inclusion and exclusion criteria

#### Inclusion criteria

1. Voluntary spontaneous delivery.
2. American Society of Anesthesiologists (ASA) classification II, indicating mild systemic disease with no functional limitations (e.g., well-controlled hypertension, uncomplicated diabetes mellitus).
3. Age > 18 years.
4. Full-term pregnancy with imminent labor.
5. No severe cardiovascular or cerebrovascular disease.
6. Adequate communication skills to participate in the study.
7. Voluntary participation and signed informed consent.

#### Exclusion criteria

1. Women who required conversion to cesarean section during labor.
2. Unstable psychiatric conditions or pre-existing depressive disorders.
3. Severe cardiovascular or cerebrovascular disorders.
4. History of ketamine or substance abuse.
5. Chronic use of medications within 6 months prior to the study.
6. Chronic alcohol abuse.
7. Inability to communicate effectively.
8. Refusal to provide written informed consent.

### Group allocation and intervention

Participants were recruited during hospitalization for labor by obstetricians who provided detailed information about the analgesia options and the study procedure. After ensuring that participants fully understood their rights and the study details, randomization was performed using computer-generated random numbers (SPSS 15.0) by an independent statistician, who was not involved in the clinical trial. Block randomization with a block size of 6 was used to allocate participants into three groups with a 1:1:1 ratio. The allocation sequence was pre-determined and sealed in opaque envelopes, which were opened only after participant enrollment.

The three groups were defined as follows:

Control Group (Group C): No labor analgesia.
Experimental Group A (Group A): Participants received labor analgesia combined with esketamine. Esketamine (0.5 mg/kg; Hengrui, Jiangsu, China) was added to the 100 mL epidural analgesic solution. The epidural pump was programmed with the following parameters: a background infusion rate of 2 mL/h; a programmed intermittent bolus (PIEB) of 8 mL administered every hour, with each bolus infused over a duration of 1 min; and a PCEA lockout interval of 30 min. The administration was continued until the completion of delivery.
Experimental Group B (Group B): Participants received labor analgesia alone. The epidural analgesia protocol consisted of a loading dose of 0.1% ropivacaine + 0.25 μg/mL sufentanil (8 mL), followed by a patient-controlled epidural analgesia (PCEA) pump set to 8 mL bolus, 30 min lockout, and an optional background infusion of 4 mL/h.

### Data collection

To minimize the impact of circadian rhythm on hormone levels (especially cortisol), venous blood samples were collected strictly between 08:00 and 10:00 a.m. This standardized timing was applied to both the prenatal baseline assessment (at admission) and the postpartum assessment (on the morning of postpartum day 1). The levels of estrogen, progesterone, 5-HT, and cortisol were measured using ELISA kits (Mlbio, Shanghai, China). The intra-assay and inter-assay coefficients of variation were < 10% and < 15%, respectively.

Systolic blood pressure (SBP), diastolic blood pressure (DBP), heart rate (HR), and pulse oxygen saturation (SpO2) were monitored continuously during labor using a multiparameter patient monitor (Mindray, Shenzhen, China). Data were recorded at prenatal baseline, intrapartum (during active labor), and at 2 h, 1 day, and 2 days postpartum.

The EPDS was administered to participants at prenatal, postpartum (1 day, 7 days, and 42 days) time points to assess depression severity. The EPDS consists of 10 items, with a total score ranging from 0 to 30, where a score ≥10 indicates the presence of postpartum depression.

In addition, the Visual Analogue Scale (VAS) was used to assess pain at various time points, including during labor and on postpartum days 1, 2, and 7. The VAS is a continuous scale where participants rate their pain intensity from 0 (no pain) to 10 (worst possible pain).

### Safety assessment

Safety and adverse events were monitored continuously from the initiation of analgesia until 2 h postpartum. Maternal vital signs (blood pressure, heart rate, and SpO2) were recorded regularly. Participants were specifically assessed for potential esketamine-related adverse effects, including dizziness, nausea, vomiting, drowsiness, and dissociative or psychotomimetic symptoms (e.g., hallucinations, dysphoria). Any adverse events were documented by the attending anesthesiologist and obstetrician.

### Statistical analysis

Data were analyzed using SPSS 26.0 software (SPSS Inc., Chicago, IL, United States), and graphical representations were created using GraphPad Prism 8.0 software (GraphPad Software, San Diego, United States). The primary analysis was performed on a per-protocol basis, including only participants who underwent spontaneous vaginal delivery as per the original group allocation. Participants who converted to cesarean section were excluded from the final analysis. This exclusion was necessary because the physiological stress response, hormonal fluctuations, and postoperative pain trajectories associated with surgical delivery differ significantly from those of spontaneous labor, which would introduce substantial confounding factors to the primary and secondary outcomes. The normality of the data was assessed using the Kolmogorov–Smirnov test, and the homogeneity of variance was tested using Levene’s test. Categorical variables were expressed as proportions and analyzed using chi-square tests. Continuous variables were presented as means ± standard deviation (X̄ ± SD). Independent sample *t*-tests were used to compare baseline demographic and clinical characteristics between groups. For longitudinal data, Repeated measures ANOVA (or Linear Mixed Models) was used to analyze the changes in EPDS and VAS scores over time and to compare differences between groups, accounting for the repeated measurements within subjects. The Mann–Whitney U test was applied to non-normally distributed data. Analysis of variance (ANOVA) with post-hoc Tukey’s multiple comparisons was used to assess differences in hormone levels between the groups. A *p*-value of less than 0.05 was considered statistically significant.

### Follow-up

Participants were followed up at 1 day, 7 days, and 42 days postpartum for clinical assessment. Hormone levels, as well as EPDS and VAS scores, were re-measured at these follow-up time points. Data from the three groups were analyzed longitudinally and cross-sectionally to determine the effects of esketamine combined with labor analgesia on postpartum depression and pain perception.

## Results

### Demographic information

A total of 312 women were initially enrolled in the study between February 2020 and January 2022. Thirteen participants were excluded due to the need for cesarean section during labor, leaving 299 women in the final analysis. These participants were divided into three groups: Group A (*n* = 100, labor analgesia + esketamine), Group B (*n* = 100, labor analgesia alone), and Group C (*n* = 99, no labor analgesia) (Supplementary Figure S1). There were no statistically significant differences in demographic characteristics, including age, body mass index (BMI), and gestational age, across the three groups (Supplementary Table S1). The baseline characteristics of the participants were comparable between the groups.

### Comparison of EPDS and VAS scores among the groups

**Figure 1:**
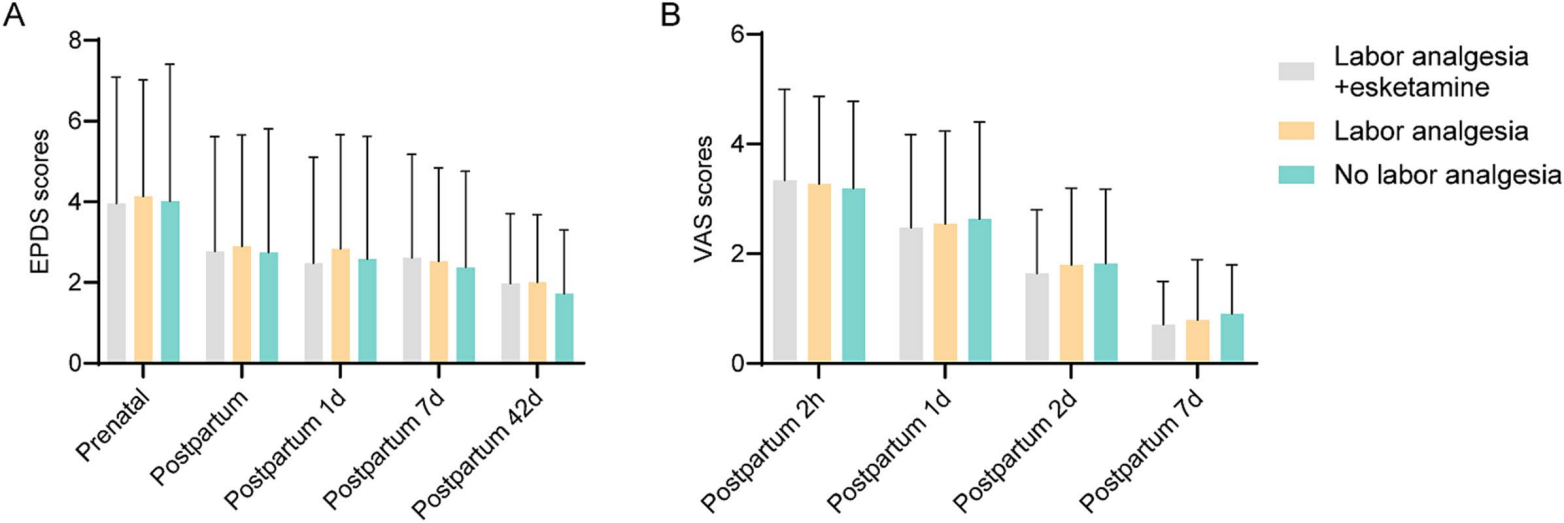
EPDS and VAS scores between each group. **(A)** EPDS scores between each group at different times, including prenatal, intrapartum, and 1, 7, and 42 days postpartum. There was no significant difference in EPDS scores between groups. **(B)** VAS scores between each group at different times including 2 h, 1 day, 2 days, and 7 days postpartum. There was no significant difference in VAS scores between groups.

The incidence of depression and the EPDS scores were compared across the three groups at multiple time points: prenatal, postpartum (1 day, 7 days, and 42 days). No significant differences in EPDS scores were observed between the groups at any of the time points assessed, including prenatally, postnatally, and at 1, 7, and 42 days postpartum (Figure 1A). Specifically, regarding the incidence of depression (defined as EPDS score ≥ 10) in the postpartum period, no statistically significant difference was found among the groups (*p* = 0.223). The incidence rates were 4.0% (4/100) in Group A, 2.0% (2/100) in Group B, and 6.1% (6/99) in Group C (Supplementary Table S2). Similarly, VAS scores, which were used to assess pain perception, did not show significant differences between the groups at any time point, including prenatal, postnatal, and on days 1 and 7 postpartum (Figure 1B).

### Comparison of serum hormone levels among the groups

**Figure 2:**
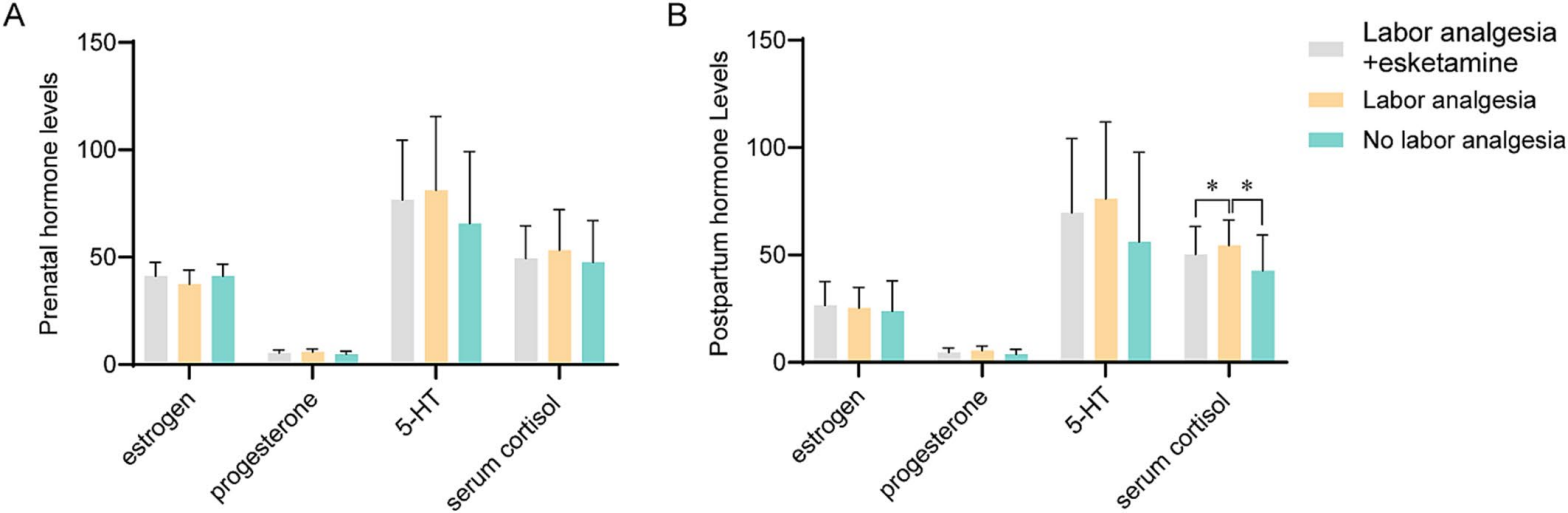
Hormone levels of prenatal and postnatal between each group. **(A)** Prenatal hormone levels in each group, including estrogen (pmol/L), progesterone (nmol/L), 5-HT (ng/mL), and serum cortisol (nmol/L). There were no significant differences in the hormones between each group. **(B)** Postnatal hormone levels in each group, including estrogen, progesterone, 5-HT, and serum cortisol. Serum cortisol levels were significantly increased in labor analgesia group as compared to other two groups. * Indicate *p* < 0.05.

Serum levels of estrogen, progesterone, 5-hydroxytryptamine (5-HT), and cortisol were compared between the three groups both pre- and post-delivery. At baseline, no significant differences in estrogen, progesterone, or 5-HT levels were found between the groups (Figure 2A). Post-delivery, no significant differences in the concentrations of these hormones were observed between the groups (Figure 2B).

However, significant differences were detected in cortisol levels. No significant differences in prenatal cortisol levels were observed between the groups. In contrast, postnatal serum cortisol levels were significantly higher in Group B (labor analgesia alone) compared to the other two groups. This suggests that a distinct stress response was elicited in women receiving labor analgesia alone, as indicated by the elevated cortisol levels. No significant differences in postnatal cortisol levels were found between Group A (esketamine + labor analgesia) and Group C (control) (Figure 2B).

### Comparison of systolic blood pressure and heart rate

**Figure 3:**
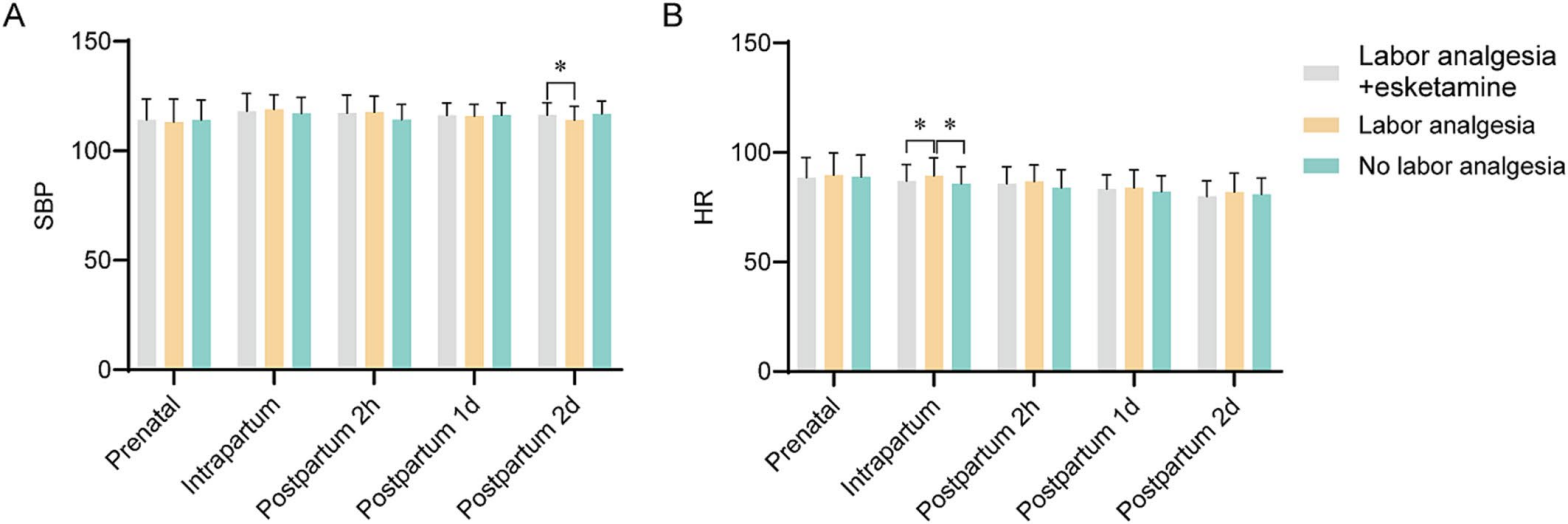
SBP and HR in each group. **(A)** SBP of each group, including antepartum, intrapartum, and postpartum 2 h, 1 day, and 2 days. At 2 days postpartum, SBP was significantly lower in the labor analgesia group than in the labor analgesia + esketamine group. **(B)** HR of each group, including antepartum, intrapartum, and postpartum 2 h, 1 day, and 2 days. HR was significantly higher in the labor analgesia group than the other two groups during delivery. * Indicate *p* < 0.05. SBP, systolic blood pressure; HR, heart rate.

Systolic blood pressure (SBP) and heart rate (HR) were measured prenatally, intrapartum, and at 2 h, 1 day, and 2 days postpartum. Across most time points, no between-group differences were detected. A significant between-group difference in SBP was observed only at 2 days postpartum, where lower SBP was recorded in the labor-analgesia group relative to the esketamine + labor-analgesia group (*p* < 0.05; Figure 3A). For HR, a significant intrapartum elevation was detected in the labor-analgesia group compared with both the esketamine + labor-analgesia and no-analgesia groups (*p* < 0.01; Figure 3B). At all other time points, HR was not found to differ significantly among groups.

### Within-group comparison of EPDS and VAS scores

**Figure 4:**
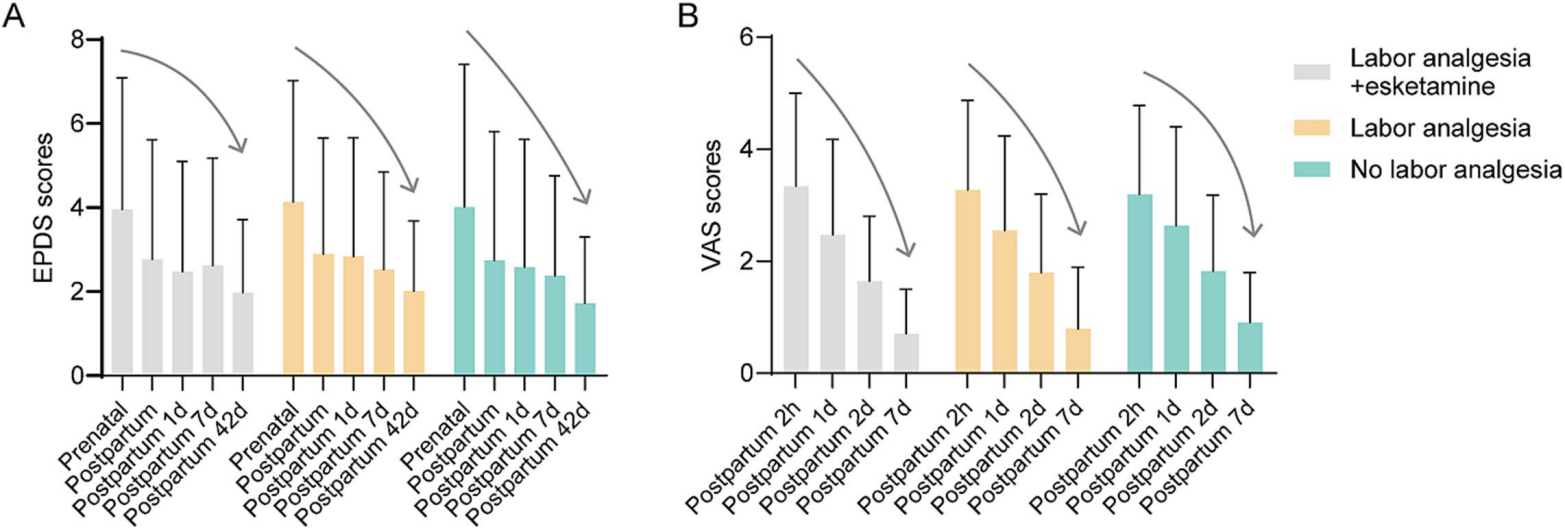
EPDS and VAS scores at different time points in each group. **(A)** EPDS scores for each group at different time points. EPDS scores were significantly lower in all three groups at the time of delivery and at 1, 7 and 42 days postpartum compared to the prenatal period and decreased progressively along with the time of delivery. **(B)** VAS scores for each group at different time points. VAS scores also decreased significantly at 1, 2 and 7 days postpartum compared to 2 h postpartum. a progressive decrease was observed along with the time of delivery. The solid black line represents a declining trend.

Within-group analyses were conducted to evaluate temporal changes in EPDS and VAS scores. In all three groups, EPDS scores were significantly lower on the day of delivery, as well as at 1, 7, and 42 days postpartum, when compared to the prenatal period (Figure 4A). This progressive decrease in EPDS scores over time suggests that an improvement in depressive symptoms occurred post-delivery in all women, regardless of treatment group.

Similarly, VAS scores showed a significant decline at 1, 2, and 7 days postpartum when compared to the 2 h postpartum time point (Figure 4B). This suggests that pain perception also decreased over time in all groups, in line with the natural recovery process following childbirth.

### Comparison of serum hormone levels within the groups

**Figure 5:**
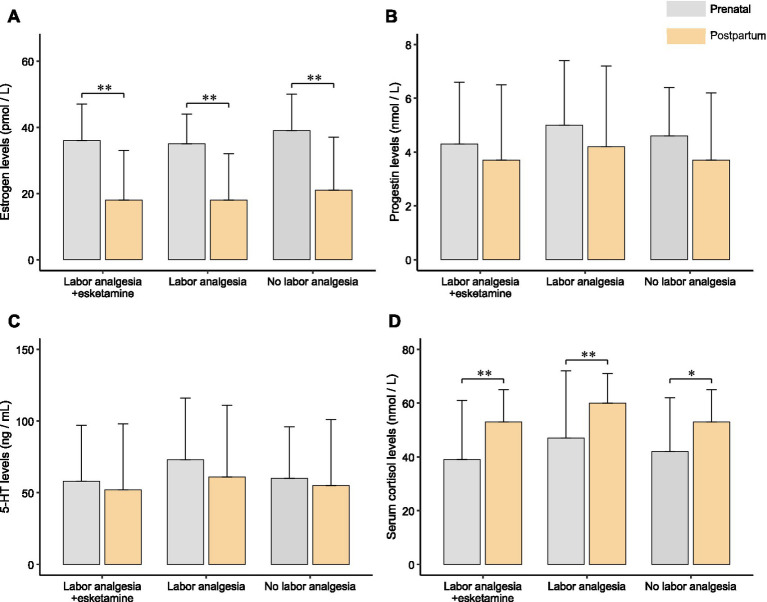
Hormone levels of the three groups during prenatal and postnatal periods. **(A)** Estrogen levels of the three groups in prenatal and postnatal periods. Estrogen levels decreased significantly after delivery in all three groups. **(B)** Progestin levels of the three groups in prenatal and postnatal. No significant changes were found in the three groups. **(C)** 5-HT levels of the three groups in prenatal and postnatal. Significant changes were not found in all three groups. **(D)** Serum cortisol levels of the three groups in prenatal and postnatal. Serum cortisol was significantly elevated postpartum in all three groups. * Indicate *p* < 0.05; ** indicate *p* < 0.01.

The changes in serum hormone levels within each group were assessed over time. Estrogen levels were found to decrease significantly postpartum in all three groups (Figure 5A). No significant changes in serum progesterone or 5-HT levels were observed before and after delivery (Figures 5B,C). However, cortisol levels increased significantly in all three groups postpartum (Figure 5D).

### Comparison of blood pressure, heart rate, and oxygen saturation within the groups

**Figure 6:**
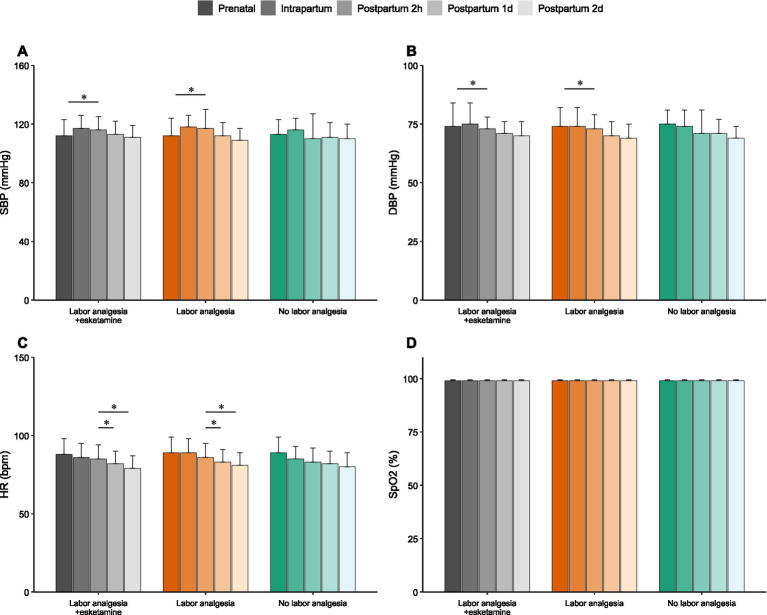
Blood pressure, heart rate and oxygen saturation at different time points in the three groups. **(A)** SBP for each group at different points in time, including prenatal, intrapartum, and postnatal 2 h, 1 day, and 2 days. **(B)** DBP for each group at different points in time, including prenatal, intrapartum, and postnatal 2 h, 1 day, and 2 days. **(C)** HR for each group at different points in time, including prenatal, intrapartum, and postnatal 2 h, 1 day, and 2 days. **(D)** SpO2 for each group at different points in time, including prenatal, intrapartum, and postnatal 2 h, 1 day, and 2 days. * Indicate *p* < 0.05; ** indicate *p* < 0.01. SBP, systolic blood pressure; DBP, diastolic blood pressure; HR, heart rate; SpO2, percutaneous arterial oxygen saturation.

Blood pressure, heart rate, and oxygen saturation were assessed within each group at different time points. Systolic and diastolic blood pressures were elevated postpartum compared to prepartum levels in both Group A and Group B, with a gradual decrease observed from 2 h postpartum (Figures 6A,B). No significant changes in heart rate were observed within Group A or Group B between the prenatal and postnatal periods. However, HR was significantly higher at 2 h postpartum compared to days 1 and 2 postpartum (Figure 6C). No significant changes in SpO2 were observed across any time point within the groups (Figure 6D).

### Safety outcomes

Maternal and neonatal safety outcomes are summarized in Supplementary Table S3. Regarding maternal adverse events, the incidence of transient dizziness was higher in Group A (12.0%) compared to Group B (2.0%) and Group C (0.0%) (*p* < 0.05). These symptoms were mild, transient, and resolved spontaneously without medical intervention. No significant differences were observed in the incidence of nausea, vomiting, or dissociative symptoms (hallucinations) among the three groups. Regarding neonatal outcomes, there were no significant differences in Apgar scores at 1 min and 5 min, or in the rates of NICU admission across the groups.

## Discussion

PPD is a prevalent psychiatric condition that affects a significant number of women worldwide. Prevalence rates are reported between 6 and 13% in high-income countries, 21% in middle-income countries, and as high as 26% in low-income regions (30). Maternal perinatal depression is often accompanied by anxiety, which impairs partner relationships and reduces mother-infant bonding compared to non-depressed mothers (31). Furthermore, children of mothers with PPD are at an elevated risk for behavioral and emotional challenges, as well as potential long-term psychological and developmental impairments (32). The underlying factors of PPD are multifactorial, including compromised physical health, restricted social support, low socioeconomic status, educational limitations, and exposure to violence (3). Despite extensive research, universally accepted predictive and treatment strategies for PPD remain elusive.

In the current study, the incidence of depression was compared across three groups—women receiving labor analgesia combined with esketamine, those receiving labor analgesia alone, and those in a control group without analgesia. The goal was to better understand the endocrine mechanisms involved in the onset of PPD. Participants who met the criteria for voluntary natural childbirth and were classified as ASA grade II were included, while women who required emergency or elective cesarean sections were excluded to ensure homogeneity and clinical relevance in the findings.

Our analysis of serum hormone levels revealed a significant reduction in estrogen and a substantial increase in cortisol levels across all three groups. These hormonal changes can be attributed to the expulsion of the placenta and the abrupt decline in hormone production at the end of pregnancy. The physiological stress incurred during labor likely triggered an increase in cortisol levels—a common hormonal response associated with stress (33). However, no significant changes in serum progesterone and serotonin levels were found before and after delivery. The stability of progesterone levels suggests a relatively balanced hormonal transition from the luteal to the menstrual phase during delivery (34). The absence of significant alterations in serotonin levels may indicate that the physiological changes during childbirth have limited impact on the serotonin system. Alternatively, this lack of change could also suggest the need for a larger sample size to detect such variations. Furthermore, several factors, such as the unique characteristics of the study population, differences in delivery methods, or the timing of hormone measurements, may have influenced these results.

Interestingly, women in Group B (labor analgesia alone) exhibited significantly higher postnatal cortisol levels compared to the other two groups. This suggests that labor pain induced a more substantial physiological stress response in this group, as indicated by the elevated cortisol levels. This finding may reflect a heightened stress response in the absence of additional pharmacological support, which could influence postpartum depression outcomes.

Although no significant changes in serotonin (5-HT) levels were observed, it is important to consider that the timing of serum sampling may not have captured transient fluctuations in serotonin, which could have been more apparent at other time points. Additionally, individual variability in hormone responses to childbirth could explain the lack of significant changes in serotonin levels. Therefore, further studies should examine hormone dynamics at additional time points to better understand their relationship with PPD.

The longitudinal analysis revealed a significant reduction in EPDS and VAS scores across all groups over time. This suggests a general improvement in both depression and pain perception following childbirth, irrespective of the treatment received. These findings align with previous studies demonstrating a natural decrease in postpartum depression scores over time (35). While no significant differences in EPDS and VAS scores were observed between the groups, it is noteworthy that the highest cortisol levels were seen in women who received labor analgesia alone (Group B). This may reflect an enhanced stress response in this group, possibly due to the pain experienced during labor. These findings suggest that labor pain and the associated physiological responses can influence postpartum depression outcomes.

Although the treatments did not lead to significant group-specific effects, all groups showed significant improvements over time. Our results showed low EPDS scores across all groups, with a very low incidence of screen-positive depression. This suggests a “floor effect,” where the baseline risk in this healthy, spontaneous labor cohort was too low to demonstrate a preventive benefit of esketamine. Future studies should target high-risk populations (e.g., women with a history of depression) to better evaluate the potential efficacy of the intervention. Future studies should explore whether more sensitive measures or additional interventions could uncover smaller, clinically relevant differences that might be missed with the current methods.

The present study does have some limitations. First, the sample size was relatively small, and the study cohort was restricted to women from a single institution in China. As such, the results may not be fully generalizable. Broader, multicenter studies with more diverse populations are needed to verify these findings. Second, although the Edinburgh Postnatal Depression Scale (EPDS) is a widely used tool for assessing PPD, it remains a subjective measure. More objective metrics, such as biomarkers or neuroimaging techniques, are needed to corroborate the findings. Third, while PPD typically emerges within the first month postpartum, this study only assessed hormonal levels at prenatal and postnatal time points, with no extended follow-up period. This limits our ability to assess the long-term effects of labor analgesia and esketamine on PPD. A major limitation of this study is the lack of blinding. Due to the distinct administration routes and clinical protocols, participants and clinicians were aware of the group assignments. This open-label design may introduce bias, particularly in self-reported measures such as EPDS and VAS scores. Future studies should employ a double-blind design with placebo controls to validate these findings.

While no serious adverse events were reported in this study, a higher incidence of transient dizziness was observed in the esketamine group, which is consistent with the known side effect profile of the drug. However, it is important to note that the use of esketamine in clinical settings can be associated with side effects, such as dissociative symptoms or transient cognitive impairments. Future studies should closely monitor and document any potential adverse effects to ensure the safety of esketamine, particularly when used in combination with labor analgesia. While esketamine shows promise for depression, its use in obstetrics must balance efficacy with safety. As noted by Di Vincenzo et al., distinguishing evidence-based efficacy from clinical myths is crucial when applying such potent psychoactive agents (28). Additionally, emerging research by Martiadis et al. suggests gender-specific responses to esketamine, particularly concerning suicidal and self-harming ideation (29). Although studied primarily in general psychiatry, these findings highlight the need for female-specific data in the peripartum period to fully understand the drug’s profile in this population. Furthermore, the follow-up period in this study was limited to 42 days postpartum, which does not allow for a comprehensive understanding of the long-term effects of esketamine on postpartum depression. Future studies should consider extending the follow-up period to assess the sustained efficacy of treatments and their potential long-term impact on maternal mental health.

In conclusion, while the addition of esketamine to labor analgesia did not significantly reduce EPDS or VAS scores in this study, a marked improvement in both depression and pain perception was observed over time in all groups. The observed hormonal changes (estrogen decline, cortisol rise) coincided with symptom improvement, but we caution against inferring a direct causal link without mediation analysis. Furthermore, the higher cortisol observed in Group B may reflect unmitigated labor stress, though notably, this did not translate to significantly higher depression scores in our cohort. Future studies should aim to refine the study design, include larger sample sizes, and extend the follow-up period to more accurately assess the efficacy of esketamine in preventing or treating postpartum depression.

## Data Availability

The original contributions presented in the study are included in the article/Supplementary material, further inquiries can be directed to the corresponding authors.
