# Supplementary material for: Effect of low-dose esketamine combined with labor analgesia on postpartum depressive symptoms following spontaneous labor: a randomized controlled trial
Source: Front Med (Lausanne). 2026 Feb 19;13:1722131. doi: 10.3389/fmed.2026.1722131 (PMC12960550; doi:10.3389/fmed.2026.1722131)
Supplement: Supplementary file 1 [file Data_Sheet_1.PDF]

Supplementary information

Effect of low-dose esketamine combined with labor analgesia on postpartum depression in spontaneous labor: a prospective cohort study

Supplemental Data:
1 Figure
2 Tables

Supplemental Figure

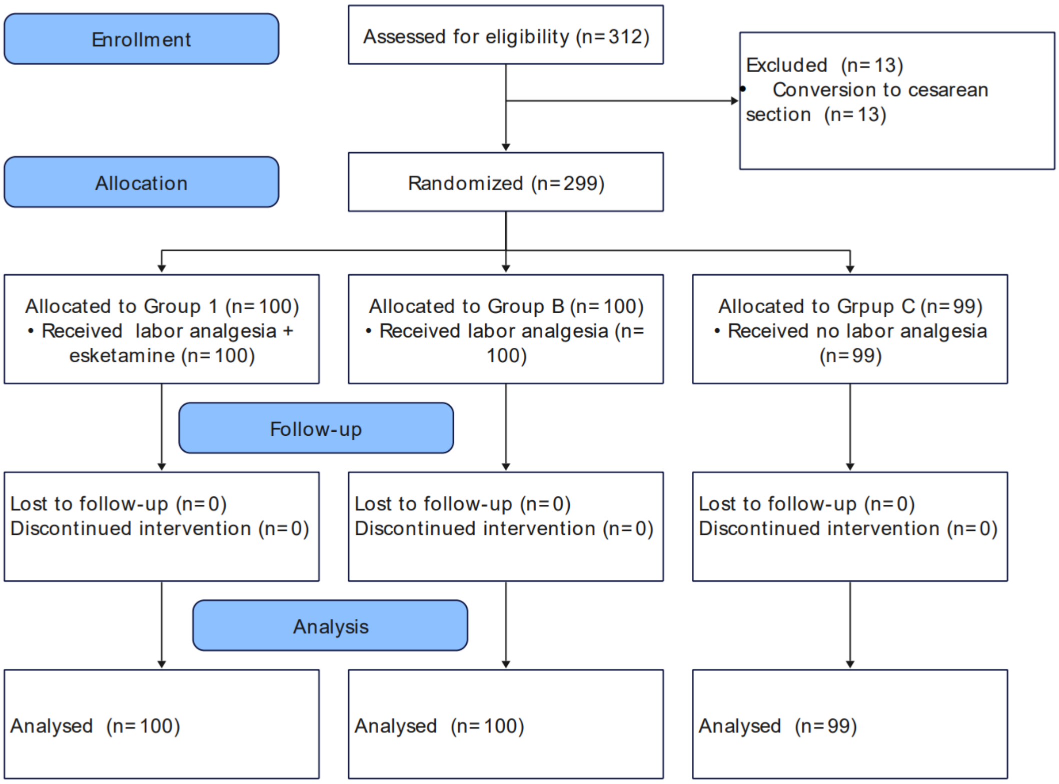

Figure S1. Design and flowchart of this study.

Supplemental Tables

Table S1. Table of patient subgroups and baseline information

Groups	Group A（n=100）	Group B（n=100）	Group C（n=99）
Age (year, `X±S）	27.1±4.9	25.9±5.1	27.2±5.4
BMI (kg/m^2, `X±S）	27.3±6.7	27.7±6.5	27.2±6.4
ASA grading (cases, II)	119	129	52
Duration of labour (min, `X±S）	244±33	236±44	250±52
MAP(mmHg,X±S)	98.0±15.7	98.6±13.9	99.1±14.6
HR(beats per minute,X±S)	75±18	78±21	74±20
Primipara/Multipara(n/n)	71/45	79/53	31/20
Weeks of Gestation(week,X±S)	39.2±1.2	39.2±1.1	39.1±1.3
Apgar(scores,X±S)	9.9±0.3	9.9±0.2	9.9±0.1
Birth fetal weight(g,X±S)	3135±411	3140±389	3149±405
Pregnancy complications (cases, n)	0	0	0

Group A: Labor analgesia combined with esketamine group; Group B: Labor analgesia alone group; Group C: Control group. BMI: Body Mass Index; ASA: American society of Aneshesiologists.

Table S2. Comparison of the incidence of postpartum depression among the three groups at different time points

Groups	Group A	Group B	Group C	χ^2	*P*-value
Prenatal	4/100 (4.0%)	7/100 (7.0%)	10/99 (10.1%)	2.731	0.255
Poatpartum	4/100 (4.0%)	2/100 (2.0%)	6/99 (6.01%)	2.7	0.223
Poatpartum 1d	4/100 (4.0%)	3/100 (3.0%)	5/99 (5.1%)	1.693	0.479
Poatpartum 7d	3/100 (3.0%)	1/100 (1.0%)	0 (0.0%)	1.667	0.396
Poatpartum 42d	4/100 4.0%)	8/100 (8.0%)	5/99 (5.1%)	1.854	0.298

Group A: Labor analgesia + esketamine; Group B: Labor analgesia; Group C: No labor analgesia

Table S3. Maternal and Neonatal Safety Outcomes

Outcomes	Group A (n=100)	Group B (n=100)	Group C (n=99)	P-value
**Maternal Adverse Events, n (%)**				
Nausea	0 (0.0%)	0 (0.0%)	0 (0.0%)	>0.99
Vomiting	0 (0.0%)	0 (0.0%)	0 (0.0%)	>0.99
Dizziness	12 (12.0%)*	2 (2.0%)	0 (0.0%)	0.01*
Dissociation / Hallucinations	0 (0.0%)	0 (0.0%)	0 (0.0%)	>0.9
**Neonatal Outcomes**				
Apgar score at 1 min (Mean ± SD)	9.9 ± 0.3	9.9 ± 0.2	9.9 ± 0.1	0.85
Apgar score at 5 min (Mean ± SD)	10.0 ± 0.0	10.0 ± 0.0	10.0 ± 0.0	>0.99
NICU admission, n (%)	0 (0.0%)	0 (0.0%)	0 (0.0%)	>0.99
